# A Circuit-Based Neural Network with Hybrid Learning of Backpropagation and Random Weight Change Algorithms

**DOI:** 10.3390/s17010016

**Published:** 2016-12-23

**Authors:** Changju Yang, Hyongsuk Kim, Shyam Prasad Adhikari, Leon O. Chua

**Affiliations:** 1 Division of Electronics Engineering, Intelligent Robot Research Center, Chonbuk National University, Jeonbuk 54896, Korea; ychangju@jbnu.ac.kr (C.Y.); shyam.rvision@hotmail.com (S.P.A.); 2 Department of Electrical Engineering and Computer Sciences, University of California, Berkeley, CA 94720, USA; chua@berkeley.edu

**Keywords:** software-based learning, circuit-based learning, complementary learning, backpropagation, RWC

## Abstract

A hybrid learning method of a software-based backpropagation learning and a hardware-based RWC learning is proposed for the development of circuit-based neural networks. The backpropagation is known as one of the most efficient learning algorithms. A weak point is that its hardware implementation is extremely difficult. The RWC algorithm, which is very easy to implement with respect to its hardware circuits, takes too many iterations for learning. The proposed learning algorithm is a hybrid one of these two. The main learning is performed with a software version of the BP algorithm, firstly, and then, learned weights are transplanted on a hardware version of a neural circuit. At the time of the weight transplantation, a significant amount of output error would occur due to the characteristic difference between the software and the hardware. In the proposed method, such error is reduced via a complementary learning of the RWC algorithm, which is implemented in a simple hardware. The usefulness of the proposed hybrid learning system is verified via simulations upon several classical learning problems.

## 1. Introduction

Artificial Neural Networks (ANNs) have been implemented successfully and achieved great success recently [1,2,3]. However, they are mostly based on a software version of digital technology with the help of a huge number of GPUs. The system is bulky, and power consumption is very high. Therefore, the hardware implementation of artificial intelligence is still strongly required in many applications, like mobile devices. The merits of the hardware-based neural networks over those of the software-based ones are processing speed and power consumption [4,5]. The fast processing of the hardware-based neural network is due to its massively-connected analog circuit-based parallel processing. Furthermore, its low power consumption is due to pulse-based short active processing time [5]. However, the fabrication difficulty of massive analog artificial synapses is still the road block of the hardware implementation of neural networks.

Synapses are functionally the most important parts in neural networks, for which inputs are weighted by some adjustable values. The off-the-shelf electronic devices that are applicable for the construction of synapses are resistors [6], capacitors [7] and floating gate transistors [8]. However, resistors lack programmability once they are fabricated. Capacitors are widely-used devices as information storage. However, the lifetime of stored information is very short due to charge leakage. For the implementation of the multiplication function of synapses, floating gate transistors have been used, but they suffer from high nonlinearity in synaptic weightings. Therefore, none of these devices can be considered as a candidate for the implementation of synapses. Recently, a new element called memristor has appeared and opens up new horizons [9,10] for the implementation of synapses. It has the feature of nonvolatile programmable memory. Once the memristor is programmed with an applied voltage or current, the information is kept until the next input voltage or current is applied. Another feature of the memristor is analog multiplication. If an input signal is represented in current mode and its multiplicand is resistance in a memristor, multiplication between them is obtained as a voltage form according to Ohm’s law.

(1) *v* = *i* × *M*

The fact that an analog multiplication is achieved with a single element of a memristor is a very remarkable merit. For these reasons, the memristor has been known as the most promising candidate for synapse implementation [11]. Snider presented a memristor-based implementation of Spike-Timing-Dependent Plasticity (STDP) [12]. Kim et al. proposed a signed artificial synapse with a memristor bridge architecture [4,5,13].

As well as the processing part of neural networks discussed above, a learning algorithm has to be implemented in the neural network system. The Backpropagation (BP) algorithm is regarded as the most powerful learning algorithm of neural networks [14,15]. However, the algorithm is involved in a huge amount of multiplications, sums and nonlinear functions. Furthermore, the fact that the measurement of the weights and the states of all nodes are required at every iteration is a very big burden. The difficulties are escalated when imperfections and mismatches are involved in the fabrication of circuit components. 

Some researchers avoided the implementation difficulty of the backpropagation with the help of software called the chip-in-the-loop learning algorithm (CIL) [16,17], where complicated arithmetic required for the backpropagation algorithm is performed in software by a host computer, and updating values are downloaded on the hardware version of neural networks at every iteration. Though the implementation burden of the complicated circuitry of the BP algorithm is reduced in this approach, the communication load for reading out the states and weights of the neural network circuit at every iteration is very heavy.

A slightly different approach from above is proposed also by [18], where learning is completed in software. After that, learned parameters (weights) are downloaded and programmed on the hardware version of the neural network. However, there is no consideration for the error caused from the difference between the software version and its hardware version of neural networks. 

Other groups of researchers proposed a different kind of learning rule, which is easier for hardware implementation [19,20,21]. Instead of using gradient descent directly, these algorithms utilize an approximation of the gradient, which is much easier for hardware implementation. The Random Weight Change (RWC) algorithm [22] is one of the representative learning algorithms belonging to this category. The weight of each synapse is changed randomly by a fixed amount at each iteration. Only the weight changes with which error is reduced are taken for updating the weights. Therefore, the learning procedure is simple and easy to implement with off-the-shelf circuit components [23,24]. However, a system implementation study had not been performed fully due to the lack of devices for neural synapses at that time.

The proposed algorithm is a hybrid learning of the software-based backpropagation algorithm and circuit-based RWC learning. The software-based backpropagation is performed on the host computer firstly. Then, learned parameters (weights) are transplanted to the physical neural circuit via programming. Error created due to difference between the software and hardware versions of the neural network is eliminated via a complementary learning with the simple circuit of the RWC algorithm. Therefore, the responsibility of the error goes to the incompatibility of the parameters, which are obtained by software, but run on hardware-based neural circuits. Normally, during learning parameters with the software-based algorithm, the limitations, such as nonlinearity and limited dynamic ranges of the hardware circuits on which the parameters are to run, are not included. Some amount of error occurs inevitably whenever parameters obtained with software are transplanted on the neural hardware circuits.

The proposed approach is new, which is not addressed by any others so far. It will be utilized importantly to solve the incompatibility problem, which can be encountered often when a hardware-based neural network is to be developed.

The rest of the paper is organized as follows. Section 2 describes the memristor-based neural architecture, and Section 3 describes the proposed hybrid learning method. Then, Section 4 and Section 5 are the simulation results and the conclusion, respectively.

## 2. Memristor-Based Neural Network

Two major functions of biological neural synapses are analog multiplication and information storage. Building an analog multiplier artificially requires more than 10 transistors, which is a heavy burden for the implementation of artificial synapses per node. Though analog multiplication can be achieved with a single resistor via Ohm’s law, namely, *v* = *i* × *R*, the resistor cannot be utilized for the artificial synapse, since it is not programmable. Recently, a resistor-like, but programmable element called the memristor has been fabricated successfully and opens the horizon in this field. One weakness of the memristor to be an artificial synapse is the lack of a negative value expression. The memristor bridge synapse is developed to overcome such a weakness of the memristor [13]. It is composed of four memristors, which can provide a signed weighting and is regarded as a promising architecture for implementing synaptic weights in artificial neural networks.

### 2.1. The Memristor Bridge Synapse

**Figure 1:**
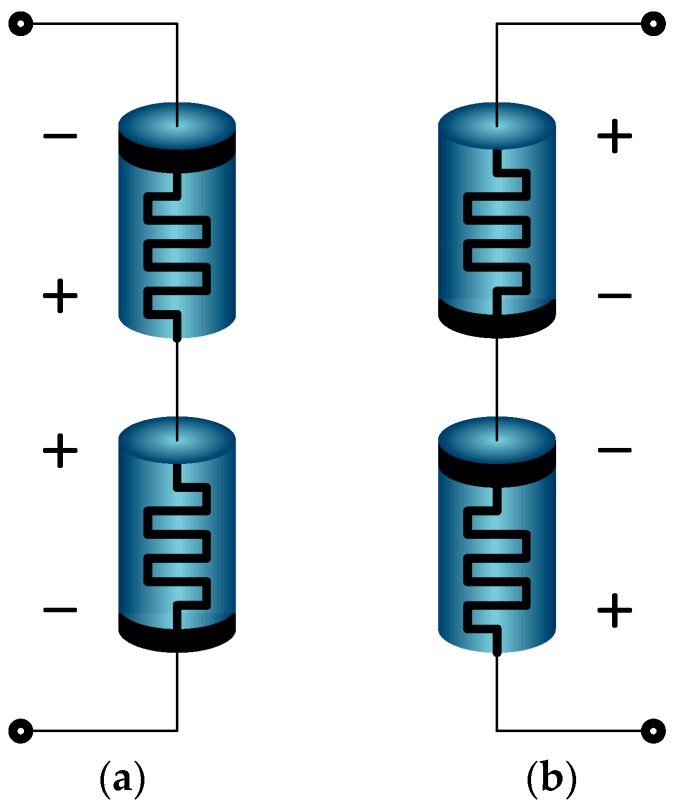
Two types of anti-serial memristor pairs. The total resistances of both cases are constant, while the voltage variations at the middle points of both cases are different. (**a**) series connection using reverse and forward direction; (**b**) series connection using forward and reverse direction.

**Figure 2:**
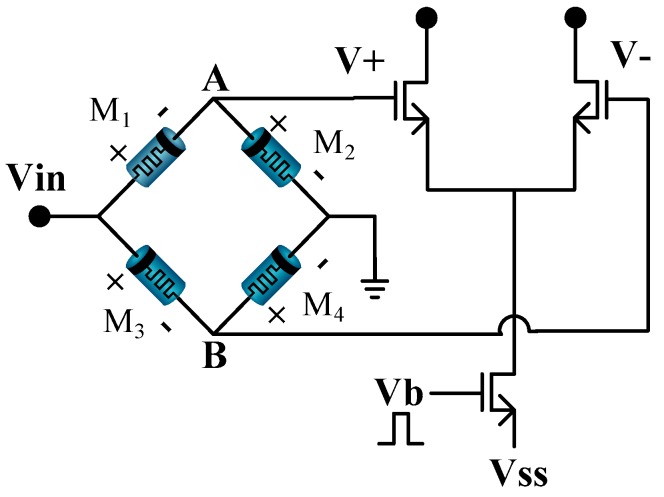
Memristor bridge synaptic circuit. The weighting operation is performed by the memristor bridge circuit, and voltage-to-current conversion is performed by the differential amplifier.

Memristance (resistance of memristor) variation is a nonlinear function of the input voltage [13]. When two identical memristors are connected in opposite polarity, total memristance becomes constant dramatically due to their complementary actions. This connection is called back-to-back connection or anti-serial connection. There are two types of back-to-back memristor (anti-serial memristor) pairs depending on the directions of polarities, as shown in Figure 1. When these two memristor pairs are connected in parallel, a bridge type synapse is built, as shown in Figure 2.

The memristor-based bridge circuit, shown in Figure 2, can be used as a synapse in the proposed NN architecture. When a positive or a negative pulse $V_{in}$ is applied at the input terminal, the memristance of each memristor is altered depending on its polarity [13]. By using the voltage divider formula, the output voltage between Nodes A and B is given as,

(2) $$V_{out}=V_{A}-V_{B}=\left(\frac{M_{2}}{M_{1}+M_{2}}-\frac{M_{4}}{M_{3}+M_{4}}\right)V_{in}$$

Equation (2) can be rewritten as a relationship between a synaptic weight ψ and a synaptic input signal $V_{in}$ as follows:

(3) $$V_{out}=\psi \times V_{in}$$

where,

(4) $$\psi =\left(\frac{M_{2}}{M_{1}+M_{2}}-\frac{M_{4}}{M_{3}+M_{4}}\right)$$

where the range of ψ is [−1.0, +1.0]. This voltage is converted to the corresponding current with the transconductance parameter $g_{m}$. The currents at the positive and the negative output terminals of the differential amplifier associated with the *k*-th synapse are:

(5) $$\begin{array}{l} i_{k}^{+}=-\frac{1}{2}g_{m}\psi^{k}V_{in}^{k}\\ i_{k}^{-}=\frac{1}{2}g_{m}\psi^{k}V_{in}^{k} \end{array}\}$$

where $i_{k}^{+}$ and $i_{k}^{-}$ are the currents at the positive and the negative terminals, respectively.

### 2.2. Memristor-Based Neural Networks

**Figure 3:**
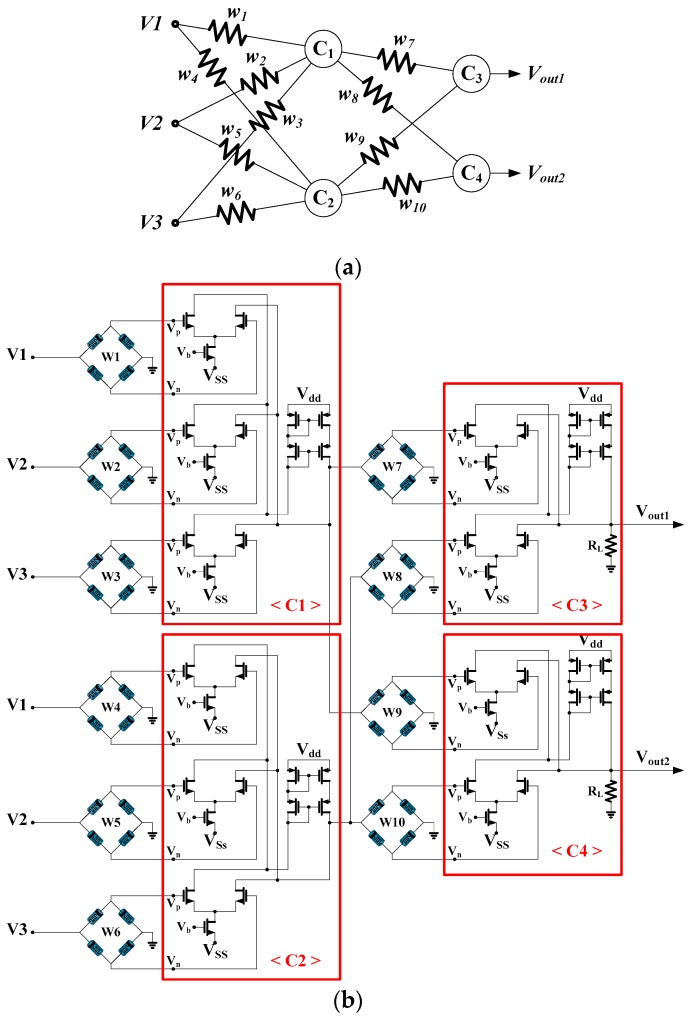
An illustration of a memristor synapse-based multilayer neural network: (**a**) a multilayer neural network; (**b**) the schematic of memristor synapse-based multilayer neural circuit corresponding to the neural network in (**a**).

Figure 3a shows a typical neural network where each neuron is composed of multiple synapses and one activation unit. The structure of a bigger neural network is simply the repeated connection of such synapses and neurons. The schematic of a memristor bridge synapse-based neuron in Figure 3a is shown in Figure 3b. In Figure 3b, voltage inputs weighted by memristor bridge synapses are converted to currents by differential amplifiers.

In the proposed circuit, all positive terminals of the input synapses are connected together, as are the negative terminals, and the sum of each signed current is computed separately. The sum of each signed current is:

(6) $$\begin{array}{l} i_{SUM}^{+}=-\frac{1}{2}\sum_{k}g_{m}\psi^{K}V_{in}^{K}\\ i_{SUM}^{-}=\frac{1}{2}\sum_{k}g_{m}\psi^{K}V_{in}^{K} \end{array}\}$$

where $i_{SUM}^{+}$ and $i_{SUM}^{-}$ are the sum of currents at the positive and the negative terminals, respectively. The output current of the active load circuit is the difference between these two current components. It follows that,

(7) $$i_{OUT}=\sum_{k}g_{m}\psi^{K}V_{in}^{K}$$

Assuming that a constant resistance $R_{L}$ is connected at the output terminal, the output voltage of the neuron is:

(8) $$V_{OUT}=R_{L}\sum_{k}g_{m}\psi^{K}V_{in}^{K}$$

The voltage at the output is not linearly proportional to the current and is soon saturated when the output voltages exceeds $V_{DD}-V_{th}$ or $V_{SS}+V_{th}$, where $V_{th}$ is the threshold voltage of the two transistors of the active load or synapses. Thus, the range of $V_{OUT}$ is restricted as follows:

(9) $$V_{SS}+V_{th}\leq V_{OUT}\leq V_{DD}-V_{th}$$

Let the minimum voltage $V_{SS}+V_{th}$ be $V_{MIN}$ and the maximum voltage $V_{DD}-V_{th}$ be $V_{MAX}$.

(10) $$V_{out}=\{\begin{array}{ccc} R_{L}I_{OUT} & \;\mathrm{if}\; & \frac{V_{SS}+V_{th}}{R_{OUT}}\leq I_{out}\leq \frac{V_{DD}-V_{th}}{R_{OUT}}\\ V_{MAX} & \;\mathrm{if}\; & \frac{V_{DD}-V_{th}}{R_{OUT}}\leq I_{out}\\ V_{MIN} & \;\mathrm{if}\; & I_{out}\leq \frac{V_{SS}+V_{th}}{R_{OUT}} \end{array}$$

**Figure 4:**
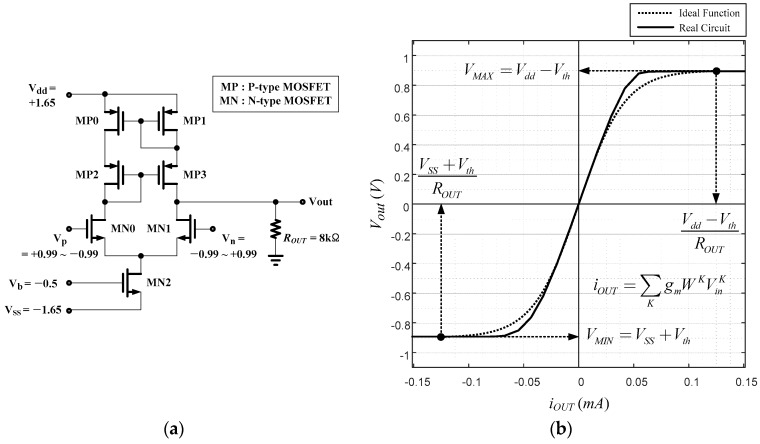
A circuit for a neural node (**a**) and its activation function (**b**).

Then, the circuit for a neural node is as in Figure 4a. The activation function is as shown in Figure 4b.

## 3. Proposed Hybrid Learning: Hardware-Friendly Error-Backpropagation and Circuit-Based Complementary Learning with RWC

### 3.1. Hardware-Friendly Error Backpropagation Algorithm

**Figure 5:**
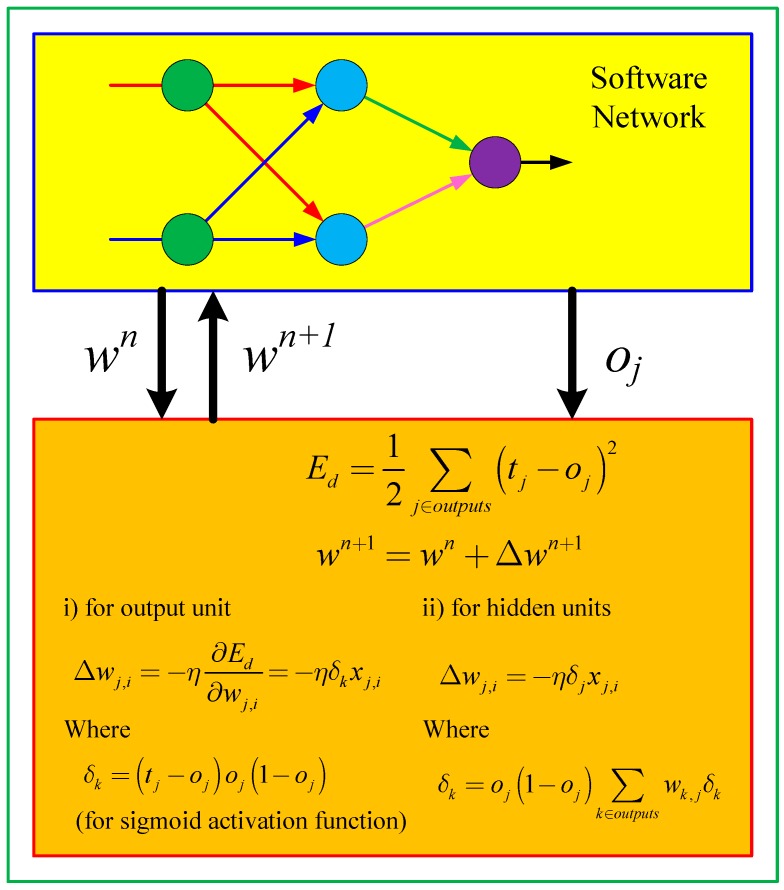
Software version of backpropagation learning for a multi-layer neural network.

The weight updating rule of the ordinary backpropagation learning algorithm [14,15] is:

(11) $$\begin{array}{cc} \Delta w_{kj}^{n} & =-\mu \frac{\partial E^{n}}{\partial w_{kj}}=\frac{\partial E^{n}}{\partial h_{k}}\frac{\partial h_{k}}{\partial net_{k}}\frac{\partial net_{k}}{\partial w_{kj}}\\ & =-\mu (t-y_{k})f\prime (net_{k})x_{j} \end{array}$$

where μ is a learning rate, *t* is a target value, $y_{k}$ is the output of an input datum, f() is an activation function and f′() is its derivative; $net_{k}$ is the summation of a weighted input value of node *k*; and $x_{j}$ is the output of previous node *j*. Figure 5 shows the software version of backpropagation learning for multi-layer neural network. 

As described in Equation (4), the weight range of the memristor-based neural network weight, $w_{circuit}$, is limited to:

(12) $$w_{output}=[-1,+1]$$

To make the software version of the neural network work well on the hardware version of the neural network, the weight value of the software version of neural networks should be limited to the same range of the circuit, namely,

(13) $$w_{software}=[-1,+1]$$

Furthermore, since the slope of the sigmoid function of the hardware version is different from that of the software version, a parameter α is multiplied to the net value. The value of α is determined, so that the slopes of the output functions of two versions are as similar as possible. 

Another arrangement is based on the issue of bipolar or unipolar output function. In the circuit design point of view, a circuit showing a nice sigmoidal shape is the one with positive and negative powers, as shown in Figure 4a. Therefore, the bipolar activation function is adopted for the output function of a node in the proposed work. 

The equation of the bipolar activation function is:

(14) $$f(net^{\prime })=\frac{1-e^{-net\cdot \alpha }}{1+e^{-net\cdot \alpha }}$$

where $net^{\prime }=net\cdot \alpha$. The summation of the weighted input of a node, *net*, in a normal bipolar sigmoid function is replaced with net·α for the adjustment of the slope. The bipolar activation function can be represented as a hyperbolic tangent function as in an activation function:

(15) $$\begin{array}{cc} f(net\prime )=h_{o} & =\tanh(net\prime )\\ & =\frac{e^{net^{\prime }}-e^{net^{\prime }}}{e^{net^{\prime }}+e^{net^{\prime }}} \end{array}$$

For the computation of the weight updating rule in Equation (11), the differentiated output function is needed. The derivative of the bipolar sigmoid function is:

(16) $$\begin{array}{cc} f^{\prime }(net\prime ) & =\frac{\partial f(net\prime )}{\partial net\prime }=\frac{\partial }{\partial net\prime }\frac{\sinh(net\prime )}{\cosh(net\prime )}\\ & =\frac{\frac{\partial }{\partial net\prime }\sinh(net\prime )\times \cosh(net\prime )-\frac{\partial }{\partial net\prime }\cosh(net\prime )\times \sinh(net\prime )}{\cosh^{2}(net\prime )}\\ & =\frac{\cosh^{2}(net\prime )-\sinh^{2}(net\prime )}{\cosh^{2}(net\prime )}\\ & =1-\tanh^{2}(net\prime )\\ & =1-f{\left(net\prime \right)}^{2} \end{array}$$

The relationship between $f^{\prime }(net)$ and $f^{\prime }(net\prime )$ can be derived with the chain rule as follows.

(17) $$f^{\prime }(net)=\frac{\partial f(net\prime )}{\partial net\prime }\frac{\partial net\prime }{\partial net}$$

Since, $\begin{array}{l} net\prime =\alpha net \end{array}$, $\frac{\partial net\prime }{\partial net}=\alpha$, Equation (17) becomes:

(18) $$f^{\prime }(net)=f\prime (net\prime )\alpha$$

Therefore, the weight updating rule in Equation (11) is:

(19) $$\Delta w_{kj}^{n}=-\mu \alpha (t-y_{k})\left(1-f{\left(net\right)}^{2}\right)x_{j}$$

**Figure 6:**
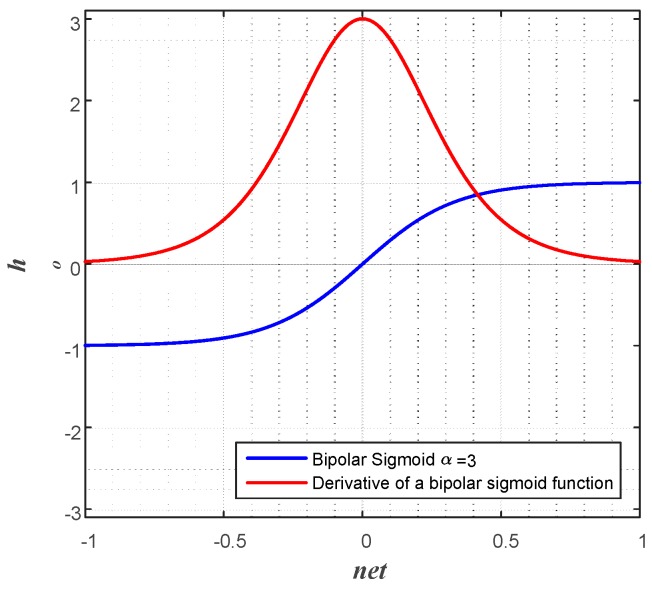
A bipolar sigmoid and its derivative functions when the multiplication factor, α, is three.

Figure 6 is a bipolar sigmoid, and its derivative functions when multiplication factor to net, α, is three.

### 3.2. Random Weight Change Algorithm for Circuit-Based Learning

**Figure 7:**
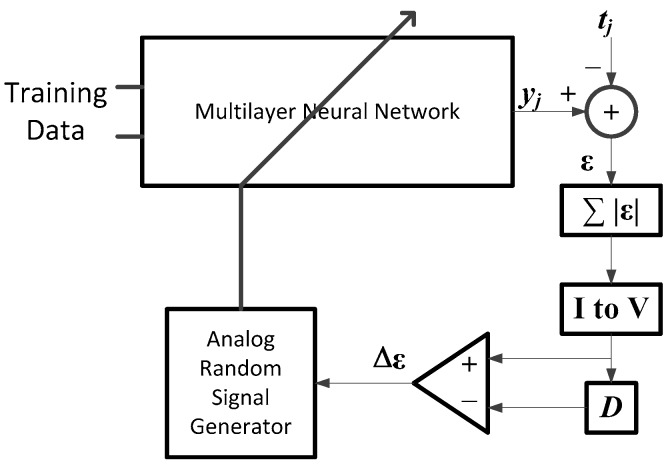
A hardware architecture of the RWC learning algorithm.

The learning algorithm for the multilayer neural network should be as simple as possible for an easy implementation with hardware as described before. In this paper, the random weight change algorithm [22] is chosen as the most adequate candidate of the neural network learning. It requires only simple circuitry, as it does not involve complex derivative calculation of the activation function, or complex circuitry for the backpropagation of error, as shown in Figure 7. It can be built by using circuit blocks for different elementary operations, like summation, square, integration, comparison and random number generation. 

Learning processing starts with a neural network programmed with random initial weights and two capacitors set with big values, which are used for the accumulation of errors in the learning loop. Then, an analog weight-updating vector (Δw) with a small magnitude is generated by an analog random weight generator and programmed additionally on the neural networks. Note that the analog random weight Δw is generated with an analog chaos generator [25] and is further simplified to ±δ, which has the same magnitude, but random signs.

After updating with the random weighting vector (Δw), an input datum $x_{j}$ is presented at the memristor-based multilayer neural networks, as shown in Figure 7, and the output $y_{j}$ of the neural network is obtained as described in Section 2. The difference between the output of the neural network and target data $t_{j}$ is computed. Then, its absolute value is taken by using an analog absolute circuit. The input signal of the analog absolute circuit is represented in current mode for the simplicity of current summation, so that the error signals in current mode are summed with a simple wired connection in the case of neural networks with multiple output terminals for the summing operation of the errors in current mode; the capacitor is used. This capacitor is also used for summing the errors of all of the training data during each iteration period. To convert the current signal back to a voltage mode, a short time current charging technique of a capacitor is adopted in this paper, where the voltage of a capacitor charging with a current *I* during time *T* can be computed via the relationship $V=\frac{IT}{C}$. 

The stored error voltage of the present iteration is compared with that of the previous iteration, which was stored in another capacitor. Note that the previous error of the first iteration is set with the biggest possible value. 

The next procedure is the updating of the weights depending on the output value of the comparator. “D” in the figure means such a capacitor, which stores the error of the previous iteration and is used for the comparison at the next iteration. If the error of the current iteration is bigger than that of the previous iteration, the updated weight vector (ΔW), which is added for the current iteration is subtracted from the weight W. Then, a new small weight vector (ΔW) is generated and added to the weight W. However, if the error of the current iteration is smaller than that of the previous iteration, the weight W is updated with the previous learning vector (ΔW), which was used for the current iteration. This learning procedure continues until the error is reduced to a small enough value. 

### 3.3. Hybrid Learning: Software-Based Confined Learning and Circuit-Based Complementary Learning with Circuits

Backpropagation is known as the most efficient learning algorithm. However, the hardware implementation of the learning algorithm is very difficult due to its complexity. On the other hand, the RWC algorithm is easy for the implementation in hardware. However, the number of required learning iterations of the RWC is very big due to its inherent random search behavior. The proposed learning algorithm is a hybrid one of these two. After learning with the software version of backpropagation, the parameter is transplanted on the physical neural circuit via programming. However, the weights obtained with software-based neural networks normally are not compatible with that of circuit-based neural networks due to the non-ideality in the implementation of hardware circuits. Programming on hardware weight is inaccurate due to the nonlinear characteristics of physical weights. For instance, weight implemented with memristor is a nonlinear function of the programming voltage. Therefore, the non-ideality in the fabrication of the circuit makes the circuit-based neural network further deviate from the theoretical model. If the hardware circuit is not identical to the software version, the error will occur significantly when the weights learned with software are transplanted on the hardware version of neural networks. Our proposed idea is the readjustment of the weight after the learned weights with the software are transplanted on the hardware-based neural network. The readjustment is performed with the hardware circuit of RWC, which is easy to implement. 

Since the location of the altered state after the learned weights are transplanted on the neural network circuit will not deviate far away from that of the software version of the neural network, the error will decrease quickly during re-learning with the RWC algorithm. 

**Figure 8:**
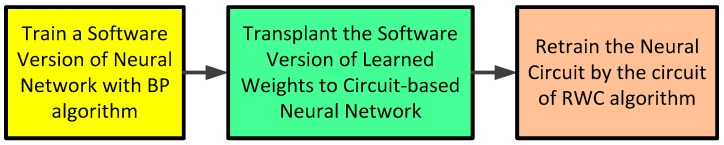
Three steps of the learning procedure of the hybrid learning of the BP and the RWC algorithms. When weights learned with the software version of a neural network are downloaded on the hardware version of neural networks, learning error increases due to the characteristic difference between software and hardware versions. The error decreases with additional learning with the RWC circuit.

Figure 8 shows there steps of the procedure of the hybrid learning of BP and RWC.

## 4. Simulation Results

The proposed research is on the hardware implementation of the neural networks and its learning system with the help of software. The hardware part, as well as the software part are implemented with software, and their simulation has been performed in a hardware described software, namely HSPICE. The synapses and nodes of neural networks are designed with the memristor bridge and differential amplifier circuits for both the software version of BP and hardware-based RWC, as described in Section 2, respectively. For the activation function of the neural node, the bipolar sigmoid function is employed.

The learning system is designed with the hardware circuit of RWC, as described in Section 3. For the simulations of the hardware part, all of the possible characteristics of the circuits are included. 

The parameters of memristors that are employed for the memristor bridge synapse are $R_{ON}$ = 400 Ω, $R_{OFF}$ = 40 kΩ, D = 10 nm and $\mu_{v}=10^{-14}m^{2}V^{-1}S^{-1}$ [9]. δ, which is used for the RWC learning, is a pulse of a one volt amplitude and width of 5 ms. It is assumed that the difference between the software and hardware versions was 10% of every parameter.

The proposed hybrid learning has been tested for three classical problems to show its effectiveness.

### 4.1. XOR Problem

A neural network with two inputs, two hidden nodes and one output node is built in a circuit to learn the solution of the XOR problem. 

**Figure 9:**
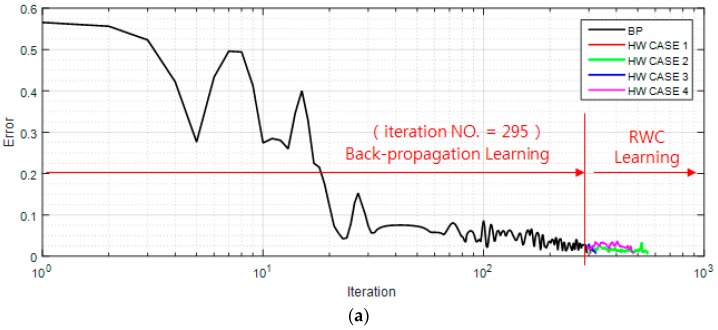

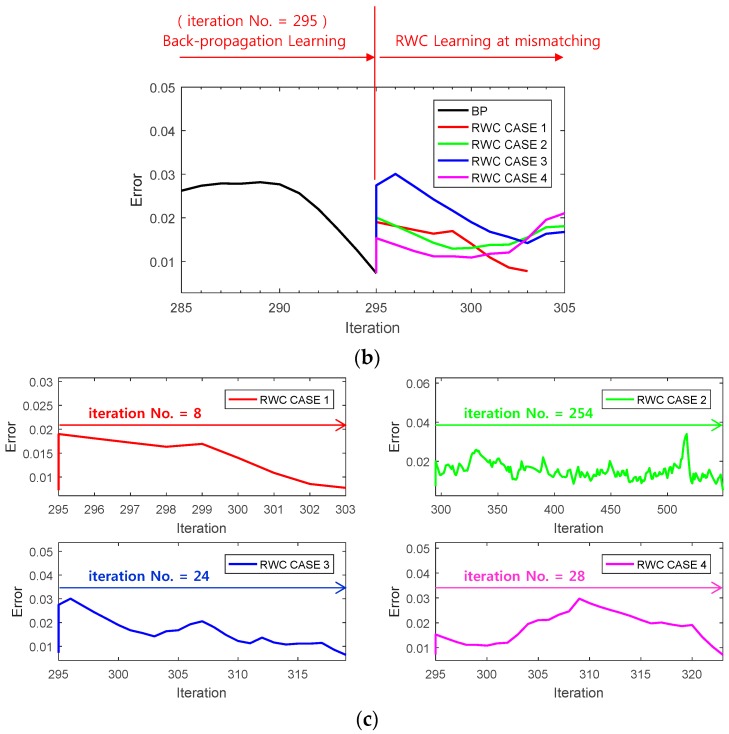
Learning curves of the XOR problem with the proposed hybrid learning: (**a**) two stages of the learning curve. After learning with BP for 295 iterations, hardware-based learning was performed with the RWC algorithm; (**b**) Magnitude graphs of the vicinity of inter-stage transition time, where learning error is increased abruptly due to the characteristics difference of software and hardware versions; (**c**) Graphs of four RWC-based learnings.

For the learning of this neural network, a backpropagation algorithm for the same network is implemented in software. Figure 9a shows a case of a learning curve of the XOR problem where the first stage of the learning is performed with the backpropagation learning algorithm until 295 iterations. Then, trained weights are transplanted on the circuit version of the neural networks, and four complementary trainings have been performed as shown at the second stage of the figure. Note that the second stage of the figure is multiple training examples with the RWC learning, while the first stage is a single training example with the BP learning. 

**Figure 10:**
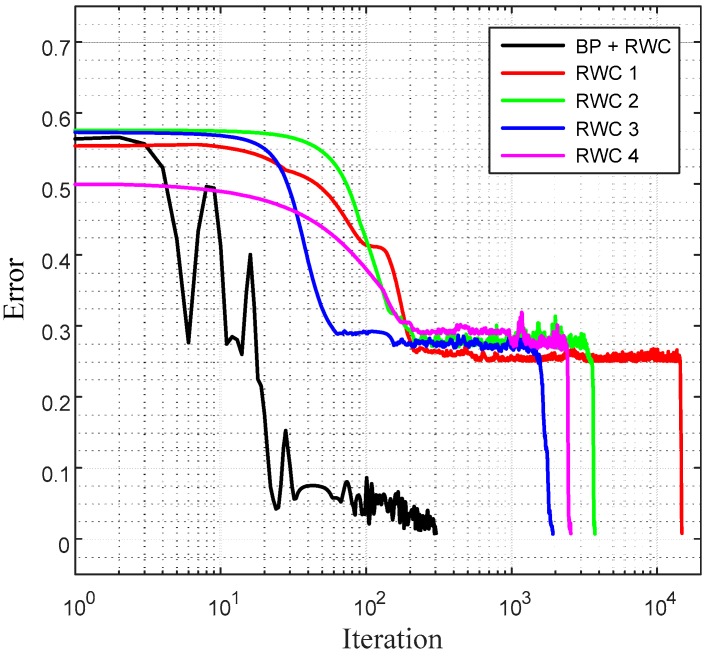
Comparison of the proposed hybrid learning and pure RWC learning for the XOR problem. The proposed learning is one order faster than that of RWC learning.

Figure 9b shows the magnified graphs of the vicinity of the inter-stage transition time. Observe that the error reduced by the first stage of learning grows abruptly after the transplanting of weights on the hardware (HW) circuit. Figure 9c shows the whole graph of the complementary learning in which learning errors of all of the learning curves are reduced successfully below the threshold, 0.0078 within 300 additional complementary iterations. Figure 10 shows a comparison of learning curves between the proposed hybrid learning and that of pure RWC learnings. Though the proposed learning is composed of two stages of learnings, such as BP and RWC, the total required learning iterations are one order less than those with pure RWC learning. Note that the horizontal axis of these figures is in log scale.

### 4.2. Three-Bit Parity Problem

**Figure 11:**
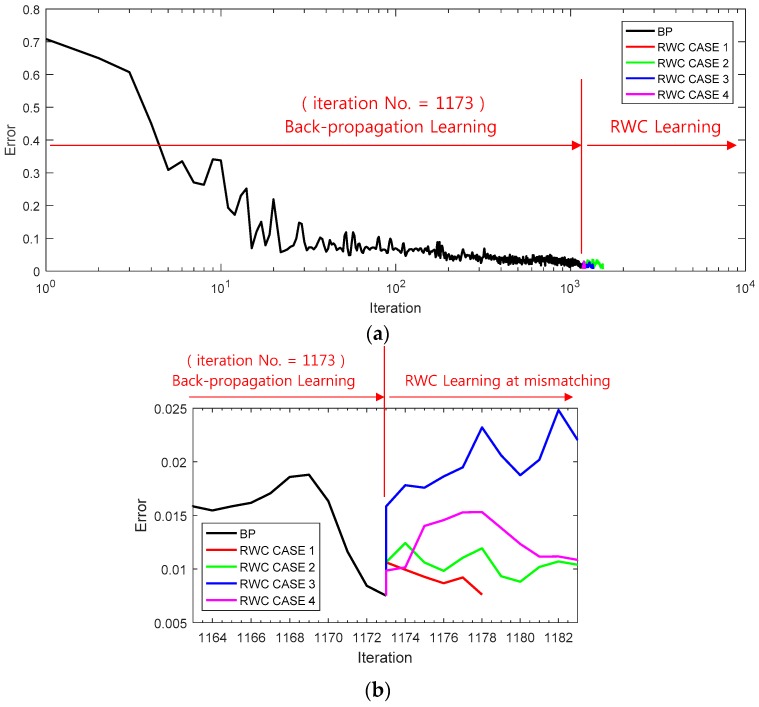

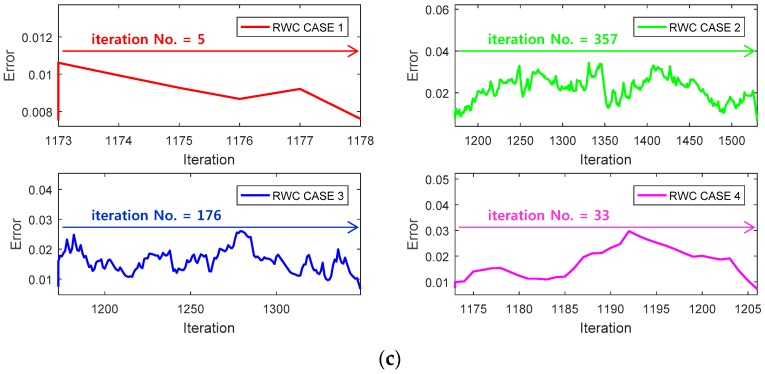
Learning curves of the parity problem with the proposed hybrid learning: (**a**) two stages of the learning curve. After learning with BP for 1173 iterations, hardware-based learning was performed with the RWC algorithm; (**b**) Magnified graphs of the vicinity of inter-stage transition time, where learning error is increased abruptly due to the characteristic difference of the software and hardware versions; (**c**) Graphs of four RWC-based learnings.

A three-bit parity problem was trained on a network of 3 input × 5 hidden × 1 output nodes. Figure 11a shows a learning curve where the first stage of the learning is performed with the backpropagation learning algorithm until 1173 iterations. Then, trained weights are transplanted on the circuit version of the neural networks, and four complementary trainings have been performed as shown at the second stage of RWC learning as in the case of the XOR problem. 

**Figure 12:**
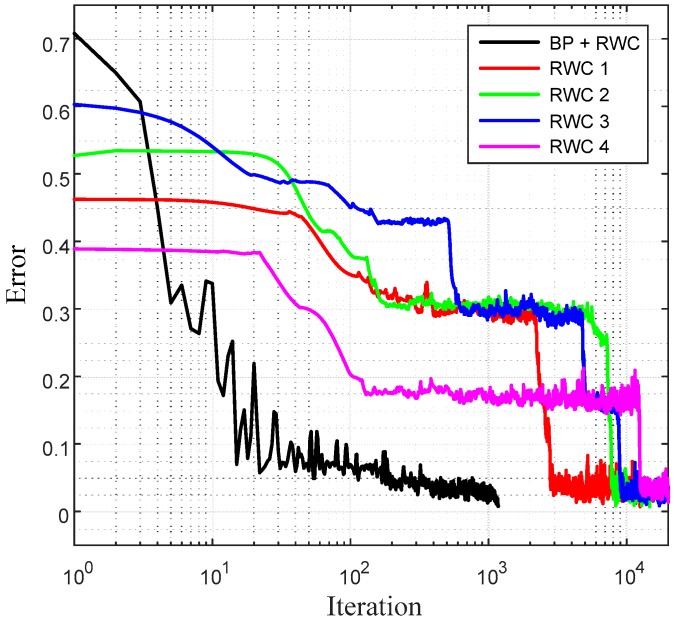
Comparison of learning curves between the proposed hybrid learning and that of pure RWC learning for the parity problem.

Figure 11b shows the magnified graphs of the vicinity of inter-stage transition time. Observe that the error reduced by the first stage of learning grows abruptly after the transplanting of weights on the hardware circuit. Figure 11c shows the whole graph of the complementary learning stage in which learning errors of all of the learning curves are reduced successfully below the threshold, 0.0078. Figure 12 shows a comparison of learning curves between the proposed hybrid learning and those of pure RWC learning. Though the proposed learning is composed of two stages of learning, such as BP and RWC, the total required learning iterations are much less than those with pure RWC learning.

### 4.3. Learning of the Robot Workspace

Another problem considered for simulating the proposed learning method is to learn the workspace of a robot. If the map of the workspace is learned by an NN, faster operations of robots without collisions with obstacles are possible. This is an important problem in robotics. After learning the map of the workspace, the robot can apply the appropriate path planning algorithm to find its way to reach the goal safely in the workspace. 

**Figure 13:**
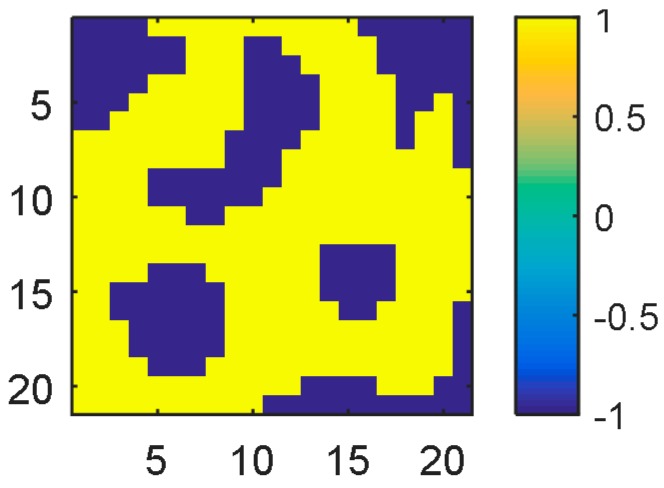
A workspace for robot navigation.

The workspace was considered as a grid, and the inputs to NN are the coordinates of the grid. The network size used for this problem was 10 input × 20 hidden × 1 output nodes. The network was trained to learn the grid of size 21 × 21, as shown in Figure 13. The coordinates in the grid without obstacles are labeled as +1 (yellow), and those with obstacles were labeled as −1 (dark blue). Each two-dimensional coordinate position in the grid was converted to a 10-dimensional binary number, i.e., the coordinate position (3, 2) in the workspace was converted to (−1−1−1+1+1, −1−1−1+1−1) to allow more degrees of freedom for learning.

**Figure 14:**
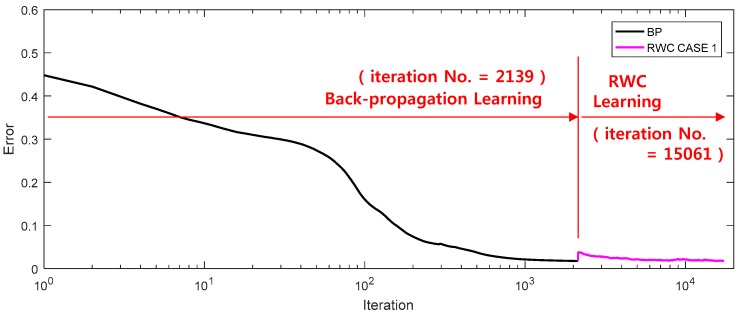
A learning curve of a robot workspace problem with the proposed hybrid learning with two stages of the learning curve. After learning with the software version of the BP algorithm, hardware-based learning was performed with the RWC algorithm. Though the error increased significantly temporally at the time of weight transplanting, it decreased by the complementary learning of RWC.

The error vs. epoch curve of the training using BP (α = 0.001) and RWC (δ = 0.00025) is shown in Figure 14. Learning of this workspace is very difficult since it is a highly nonlinear function. 

When the error reaches a threshold (0.01), the learned weights were transplanted to a neural network circuit. Then, hardware-based learning was performed with the RWC algorithm. Though the error increased significantly temporally at the time of weight transplanting, it decreased by the complementary learning of RWC. 

**Figure 15:**
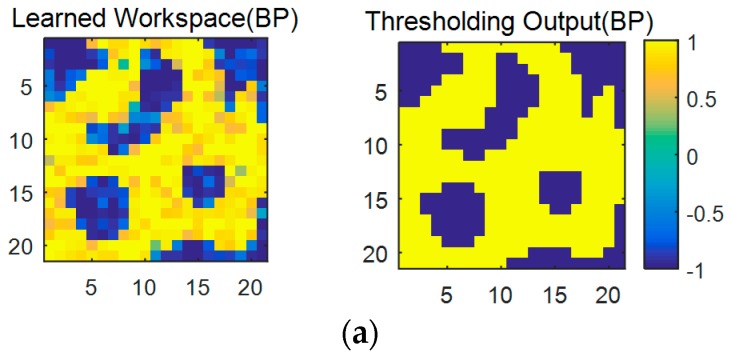

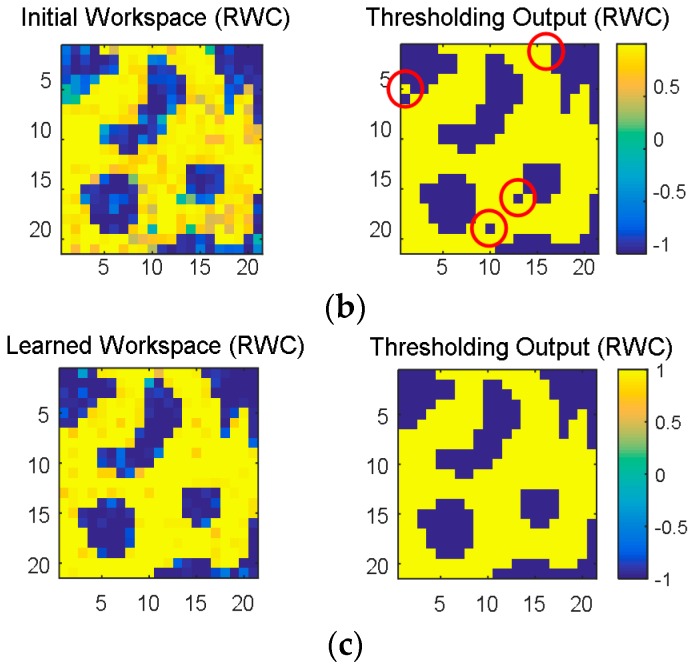
Learned results of the robot workspace with the proposed hybrid learning system. (**a**) Results with the software-based BP algorithm. The left and right ones are the ones before and after thresholding, respectively; (**b**) Outputs of the neural network circuit immediately after the learned weights are transplanted on it. Erroneous areas are marked in red circles; (**c**) Results after the complementary learning with the hardware circuit of the RWC.

Figure 15a shows the learned results of the original robot workspace in Figure 13 with the software-based BP algorithm, where the left and the right ones are the results before and after a threshold, respectively. The learned result after the threshold is identical to the original one in Figure 13. However, Figure 15b is the output of the neural network circuit immediately after the transplant of the learned weights. Observe that learning errors appear at several places (red circled area) of the right one of Figure 15b. Those errors disappear when the complementary learning with the hardware circuit of RWC is conducted, as shown in the right one of Figure 15c. This result shows the fact that the proposed hybrid learning system is a good solution for the hardware implementation of neural networks.

## 5. Conclusions

A hybrid learning method of software-based BP and hardware-based RWC is addressed in this paper. In the learning method, the software-based BP learning is conducted firstly, and its learned weights are transplanted on the neural hardware circuits. Then, the hardware-based RWC learning is continued as a complementary learning. 

For the software-based BP learning, the original algorithm is modified so that the learned weights can fit well to the neural hardware circuit; the range of weights is limited to [−1, 1], and a bipolar activation function is adopted. 

The hardware circuits of synapses and nodes are designed with memristors and differential amplifiers, respectively. The circuit of the RWC learning system is designed with off-the-shelf simple circuit elements. 

The proposed learning method has been examined with three classical problems, namely the XOR, parity and robot workspace learning problems. As expected, the error reduced with BP learning grows abruptly when weights are transplanted on the neural hardware circuit. However, after some amount of complementary learning, the error was reduced successfully below the threshold. Comparing the learning curves of the proposed hybrid learnings with those of RWC-only learning, the required learning iterations of the proposed method are much less than those with RWC-only learning. Judging with these simulation results, the proposed hybrid learning method can be a very promising solution to resolve the difficulty in the hardware implementation of neural networks.
